# The effect of low level laser irradiation on oxidative stress, muscle damage and function following neuromuscular electrical stimulation. A double blind, randomised, crossover trial

**DOI:** 10.1186/s13102-019-0147-3

**Published:** 2019-12-27

**Authors:** Ewa Jówko, Maciej Płaszewski, Maciej Cieśliński, Tomasz Sacewicz, Igor Cieśliński, Marta Jarocka

**Affiliations:** 1 Józef Piłsudski University of Physical Education in Warsaw; Faculty of Physical Education and Health in Biała Podlaska, Chair of Natural Sciences, Akademicka 2, 21-500 Biała Podlaska, Poland; 2 Józef Piłsudski University of Physical Education in Warsaw; Faculty of Physical Education and Health in Biała Podlaska, Chair of Rehabilitation, Biała Podlaska, Poland

**Keywords:** Photobiomodulation, Evoked contractions, Exercise-induced muscle damage, Biochemistry, DOMS, Knee extensors

## Abstract

**Background:**

Low level laser therapy (LLLT) is among novel methods for preventing and treating muscle damage and soreness induced by volitional exercise, but little is known about using LLLT before neuromuscular electrical stimulation. The aim of this first randomised, double blind, crossover trial addressing this issue was to evaluate effects of LLLT on muscle damage and oxidative stress, as well as recovery of muscle function after a single session of isometric neuromuscular electrical stimulation(NMES).

**Methods:**

Twenty four moderately active, healthy men aged 21–22 years received 45 electrically evoked tetanic, isometric contractions of the *quadriceps femoris*, preceded by LLLT or sham-LLLT. Maximal isometric voluntary muscle torques, perceived soreness, and blood samples were analysed from baseline to 96 h post intervention. We measured plasma markers of muscle damage (the activity of creatine kinase), and inflammation (C-reactive protein), and evaluated redox state parameters.

**Results:**

NMES-evoked contractions induced oxidative stress, demonstrated by an increase in lipid peroxidation and impairments in enzymatic antioxidant system. LLLT irradiations had a protective effect on NMES-induced decrease in enzymatic antioxidant defence and shortened the duration of inflammation. This effect of irradiations on redox state and inflammation did not affect lipid peroxidation, muscle damage, and muscle torque.

**Conclusions:**

LLLT may protect from impairments in enzymatic antioxidant system and may shorten inflammation induced by a single NMES session in moderately active, healthy men. However, the effects of LLLT on redox state and inflammatory processes do not seem to affect muscle damage and recovery of muscle function after NMES.

**Trial registration:**

The study was retrospectively registered in the Australian New Zealand Clinical Trials Registry (ANZCTR); The trial registration number: ACTRN12619000678190; date of registration: 6 May 2019.

## Background

Transcutaneous neuromuscular electrical stimulation (NMES) is considered a standard physiotherapeutic and training intervention, utilised for restoring, preserving or improving muscle strength and functional capacities [1–4]. NMES is used, in various technical and training parameters and regimes, either as a single intervention to evoke superimposed muscle activation, or else supplementary to voluntary exercise training [1, 2]. The mechanisms of voluntary and electrically evoked muscle contractions differ significantly, as regards central and peripheral mechanisms of motor unit excitation, myofibril recruitment patterns, biochemical phenomena, and fatigue [1, 4].

The specificity of electrically evoked muscle contractions might be advantageous, e.g. in synchronous excitation of myofibrils of a specific type, with the activation of fast motor units even at relatively low levels of evoked force [1]. However, the main adverse effects and potential harms include pain and discomfort [2, 3], delayed onset muscle soreness (DOMS) that may be greater than following voluntary exercise [1, 5, 6], and even excessive muscle damage [6, 7]. Direct manifestations of damage at the myofibril and sarcomere levels include macrophage infiltration, z-lines disruption, and loss in desmin immunoreactivity [8], while among indirect markers and symptoms is the increase in circulating creatine kinase (CK) activity [6, 9, 10]. Applications of NMES for muscle strengthening, despite long tradition of its use [2, 3, 11], are still being developed, including optimisation of stimulation parameters and improving comfort [3, 4]. Therefore, further studies, addressing NMES, including its use in healthy, physically active participants and athletes, are warranted. The potential mechanisms responsible for muscle damage and soreness induced by NMES, namely related to changes in prooxidant-antioxidant status, are among primary research priorities.

Among various interventions used for the prevention and treatment of exercise-induced muscle damage and DOMS [12, 13], photobiomodulation therapy (PBMT), or low level laser therapy (LLLT), is an emerging approach [14–16]. It has been indicated that LLLT preceding voluntary exercise and athletic training offers potential benefits on muscle tissue. Among many possible mechanisms potentially responsible for the above, are increases in energy metabolism and ATP synthesis, stimulation of defences against oxidative stress, and prevention or repair of muscle damage [15]. As a result, LLLT can accelerate post-exercise recovery and positively affect sports performance [15, 16]. Nonetheless, the effectiveness of the technique remains a matter of dispute, with supporting pre-clinical findings [14, 15] but with inconsistent, or even contradictory, evidence from clinical studies of varying quality [17–19]. Only a single, pilot, clinical study [20] addressed LLLT application for preventing muscle function following NMES. The authors reported that LLLT did not attenuate muscle fatigue evoked by NMES. Nonetheless, the study was performed in only five subjects, who received LLLT prior to a 3-min session of NMES of the quadriceps muscle. Therefore, those findings need to be interpreted with caution. Furthermore, volitional exercises cannot be directly extrapolated to NMES interventions [1, 6, 7, 14].

Hence, the aim of this study was to test the hypothesis that LLLT irradiations, applied prior to a single session of strenuous, tetanic, isometric NMES of *quadriceps femoris* muscle, in healthy, recreationally active men, are preventive as regards post-stimulation muscle damage, soreness, and decrease in muscle function. For that purpose, blood markers of muscle damage, inflammation, and redox state were measured in relation to muscle torques and perceived muscle soreness, in time course of the post-stimulation recovery. To the best of our knowledge, this is the first controlled trial to address this issue.

## Methods

### Study design

**Fig. 1:**
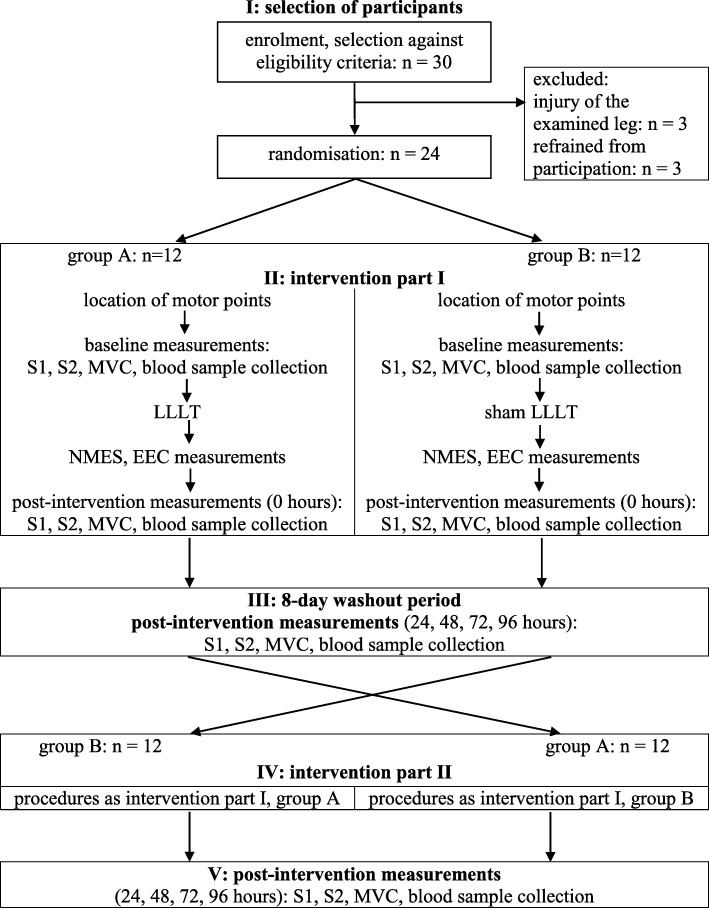
Flowchart of the study. EEC – electrically evoked contractions; LLLT – low level laser irradiation; NMES – neuromuscular electrical stimulation; MVC – maximal (isometric) voluntary contraction; S1 – pain severity measurement – pressure test; S2 – pain severity measurement –squat test

We conducted a randomised, controlled, double blind, crossover trial. In the absence of reporting guidelines for crossover studies, we consulted specific recommendations [21, 22] and the CONSORT2010 reporting guideline [23] in designing and reporting the study (Supplementary file 1). The participants were assigned to the groups through simple randomisation (tossing a coin), and the allocation was concealed. Organisation of the study is shown in Fig. 1. At the first stage (part I) of the study, group A (*n* = 12) received NMES preceded by LLLT, while group B (*n* = 12) received NMES preceded by sham LLLT. A reverse procedure was applied after an eight-day wash-out period (part II).

We assume that our study met the key requirements, critical in terms of the risk of bias in the findings and interpretation of crossover designs [21, 24, 25]. Firstly, we eliminated the carryover effect due to sufficient wash-out period. We based this assumption on our pilot investigation of the NMES intervention of identical characteristics as for the actual experiment, performed in a group of ten students, where we observed a full regaining of the baseline parameters within 8 days. Our decision was also informed by a corresponding cross-over study on LLLT for exercise performance, by leading authors in this field [26], who introduced a seven-day washout period in their trial. These assumptions were then confirmed by insignificant baseline MVC and CK differences in participants, as measured before study periods of the actual trial. Secondly, the phenomenon of period effect did not apply to our study as it was a short, single intervention in healthy individuals. There were no underlying medical conditions, hence the characteristics of the participants were stable over time and were not influenced by external, uncontrolled factors. Thirdly, we avoided drop-outs and we did not have missing data from both periods (part I and part II) of the experiment, thus we could perform full within-individual comparisons. And finally, the crossover design was appropriate in terms of the tested intervention: neither NMES nor LLLT are interventions which could permanently and systematically alter the characteristics of the participants between the two periods of the experiment.

Blinding involved both study participants and investigators, who performed experiments (M.C., E.J., T.S.) and analysed the data (I.C.). One investigator (M.J.) allocated participants to LLLT or sham LLLT.

The protocol of the study was approved by the Research Ethics Committee, University of Physical Education, Warsaw, Poland (SKE 01–14/2014), prior to the enrolment of the participants. We obtained written consents from all individual participants included in the study.

### Participants

The study included twenty four moderately active, healthy men, aged 21–22 years, with fat free mass of 19.7 ± 1.6% and body mass index of 23.7 ± 2.3 kg/m^2^ (Table 1). Sample size was determined based on methodological assumptions for crossover designs [21, 22, 24], corresponding crossover trials [17, 19, 26], and homogeneity of the group. For an assumed test power of 0.9, the minimal sample size was 23. Exclusion criteria were routine, intensive, competitive physical activity or sedentary lifestyle, lower limb injuries within a year prior to the study, current inflammation, and contraindications to NMES or LLLT [11, 27].

**Table 1:** Demographics and body composition characteristics of the participants (males, *n* = 24)

characteristics	mean ± SD / (min value – max value)
age [years]	21.9 ± 0.3 / (21–22)
body mass [kg]	78.6 ± 9.81 / (64–98)
body height [cm]	181.8 ± 4.4 / (170–190)
body mass index [kg·m^− 2^]	23.7 ± 2.3 / (20.1–27.5)
fat free mass [%]	19.7 ± 1.6 / (16.8–22.8)
fat mass [%]	4.8 ± 1.5 / (2.5–9.4)

*SD* Standard deviation, *%* Percent of total body mass

The candidates were instructed to refrain from intense physical exercise and to keep to the hygienic lifestyle during the study period. Three of the thirty subjects initially enrolled were excluded due to limb injury and three refrained from participation before the study began. There were no drop-outs throughout the study (Fig. 1).

### Measurements and procedures

#### Body composition

We evaluated body composition using Seca mBCA 515 body composition analyser, Seca GmbH&Co. KG, Germany. Self-reported physical activity was assessed at enrolment, with the use of the International Physical Activity Questionnaire (IPAQ), long form, validated national version [28], producing estimates of physical activity in four domains: work, leisure, transportation, and household. The moderate physical activity level is in the IPAQ referred to as “doing some activity more than likely equivalent to half an hour of at least moderate intensity physical activity on most days” [28].

#### Muscle strength

**Fig. 2:**
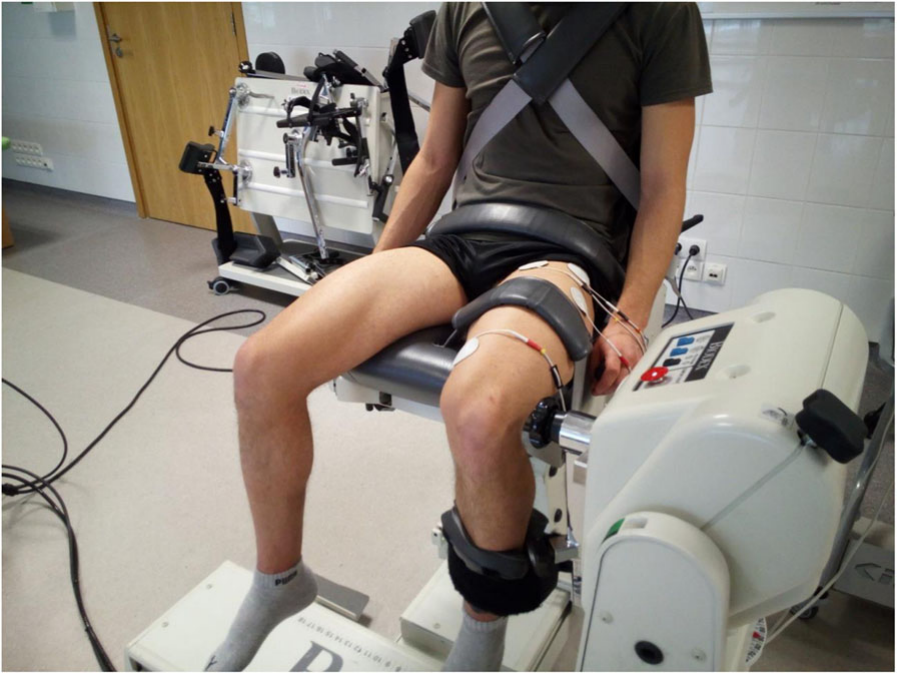
Body position and electrode placement during MVC measurements and NMES sessions

Following the enrolment, body composition measurements, and physical activity assessments, the actual experiment was carried out. The procedures were conducted in a standard manner (details were adapted from Jubeau et al. [5]), with the use of the Biodex System 4 Pro measuring device (Biodex Medical System, USA), with participants in a sitting position, with their knees bent at an angle of 100°, measured from the 0° extension of the knee joint. The participants had pelvis, thighs, and trunk stabilised with the use of straps, and they gripped stabilisation handles of the chair for full stabilisation. The dynamometer shaft axis overlapped rotational axis of the knee joint. We examined non-dominant legs (Fig. 2). Then we measured moments of force (maximal voluntary contraction in statics, MVC) of the examined knee extensors. The participants were asked to try to extend the leg at the knee joint, with possibly maximum strength, for 3 s, three times, with a 30-s. intervals. The highest values were included in the analysis.

#### Irradiations

Directly prior to NMES, we administered LLLT irradiations (based on Baroni et al. [29]) in six skin areas (25 cm^2^ each, with direct contact of the probe to skin), i.e. 5 cm above the middle of the line connecting the anterior superior iliac spine and the base of patella and 5 cm below that spot, in two areas above the *vastus medialis* belly, and in two areas above the *vastus lateralis* belly. The dose of 30 J (preventing from the thermal effects to occur) was applied in each area, with a cluster probe consisting of four semiconducting lasers, 830 nm, of 200 mW each (Table 2), using BTL 5000 device (BTL Industries Limited, London).

**Table 2:** Technical and application parameters of neuromuscular electrical stimulation and low level laser irradiation

neuromuscular electrical stimulation, NMES		low level laser, LLLT	
technical parameters:			
type of current:	pulsed, low frequency, faradic type	type of laser:	low level (low power)
pulse frequency:	80 Hz	mode/ frequency:	continuous output
pulse duration:	300 μs	wavelength:	830 nm
waveform:	square, biphasic	probe:	cluster, 4 semiconducting lasers
electrodes:	round, self-adhesive, 5 cm diameter	optical output/ power	200 mW
characteristics of intervention:			
stimulus ON time:	5 s	energy:	30 J each area
stimulus OFF time:	15 s	total energy delivered per muscle:	180 J (six areas)
ramp time:	1.0 s ON / 1.0 s OFF	mode of application:	cluster probe held stationary, skin contact with a slight pressure, 90° angle (0° angle of incidence)
number of repetitions:	45		
contraction intensity:	level of tolerance (just below pain threshold)		

The procedure of sham-LLLT intervention was identical except the light outputs of the probe were covered with aluminium foil.

#### Electrical stimulation

To locate **motor points** of the *vastus medialis* and *vastus lateralis* muscles, we induced single muscle twitches with a 0.5 cm diameter point electrode, using low frequency pulsed current (1 Hz, 300 μs), generated by Triostim device (Mettler Electronics Corporation, USA). To avoid muscle fatigue, we ceased stimulation as soon as contractions occurred, and marked a motor point with a sterile marker pen.

Then the actual NMES sessions were performed, with the use of a stationary electrical stimulator Sonicator Plus 940 (Mettler Electronics Corporation, USA), generating pulsed, two-phase, 300 μs, square-wave, symmetrical, delivered at 80 Hz, current (Table 2). Four (two electric circuits) round, self-adhesive, 5 cm diameter electrodes were placed over the motor points, onto the skin that was previously rinsed with alcohol and dried off (Fig. 2).

The NMES sessions consisted of forty five electrically evoked tetanic, isometric contractions (electrically evoked contraction, EEC), in the 5 s. ON, 15 s. OFF mode, with 1.5 s. ramp-on and 0.5 s. ramp-off times. The current intensity was set at the level of tolerance (below pain threshold) and was adjusted by an investigator (M.C.) to that level when needed. We recorded and analysed moments of force of each EEC, except first five contractions, which were excluded from the analysis, as first stimulation bouts are presumed initial and allowing the current to fully penetrate. We performed NMES based on Aldayel et al. [9] and with a typical electrical stimulation technique [11].

#### Muscle soreness

In order to assess the severity of muscle soreness, one investigator (M.C.) pressed a participant’s leg for 3 s with his fingers (1) 5 cm proximally, (2) 5 cm distally to the central area between the base of patella and anterior superior iliac spine, and over motor points of (3) *vastus medialis* and (4) *vastus lateralis* muscles, with possibly the same repeatability of force and time as well as the constancy of pressure for each attempt [9]. The participants indicated the severity of their muscle soreness on a standard 100 mm visual analogue scale with 0 corresponding to *no pain* and 100 mm to *worst imaginable pain*. Afterwards, standing with their feet spread shoulder-width apart, the subjects slowly performed a squat to 90° knee flexion and returned to the starting position. Then the muscle soreness assessment was repeated in the same manner.

#### Blood samples

We collected blood samples from the ulnar vein under fasting conditions. The samples were drawn into heparinized test tubes, then centrifuged (for 10 min. at 3.000 x g, at 4 °C) to separate erythrocytes from plasma. Subsequently, erythrocytes were washed three times with a cold isotonic saline solution. Erythrocytes, plasma, and the whole blood were frozen and stored at − 80 °C until analysis. Measured redox parameters were the activity of superoxide dismutase (SOD) in erythrocytes, the activity of glutathione peroxidase (GPx) in the whole blood, the total antioxidant capacity (TAC) of plasma, and the level of malondialdehyde (MDA) in plasma. Also, the concentration of uric acid (UA), C-reactive protein (CRP), and creatine kinase (CK) activity in plasma were evaluated. The SOD and GPx activities were determined with commercially available kits (RANSOD Cat. No. SD 125 and RANSEL Cat. No. RS 505, respectively; Randox, Crumlin, UK). The antioxidant enzyme activities were measured at 37 °C and expressed in U/g Hb. Haemoglobin (Hb) was assessed by a standard cyanmethaemoglobin method, using a diagnostic kit (HG 1539; Randox, Crumlin, UK). The TAC to scavenge ABTS radicals was measured using a chromogenic method, with commercially available kit (Cat. No. NX 2332, Randox, Crumlin, UK). Antioxidant capacity of samples was expressed as millimoles per litre of Trolox equivalents (6-hydroxy-2,5,7,8-tetramethylchroman-2-carboxylic acid). Plasma MDA level was determined based on the reaction of a chromogenic reagent, N-methyl-2-phenylindole, with malondialdehyde, at 45 °C, after the reaction with hydrochloric acid, which formed a stable chromophore with maximum absorbance at 586 nm [30]. Consequently, plasma CK activity, as well as UA and CRP concentration, were determined with the use of a diagnostic kit (Cat. No. C6512–100, K6580–200, and C6428–075, respectively; Alpha Diagnostics, Poland).

MVC measurements, muscle soreness assessments, and blood sample collections were taken prior to the NMES session (at baseline), immediately (0), 24, 48, 72, and 96 h after NMES (Fig. 1).

#### Statistical analyses

Demographics, physical activity questionnaire scores, and body composition characteristics were analysed with descriptive statistics (mean, SD, and min and max values). The corrected pairwise t-test for multiple hypotheses was used to analyse muscle strength variability, while the Kruskal-Wallis test was employed to evaluate muscle soreness. EEC values are presented as percentages of MVC. A two-way ANOVA (group, time) with repeated measures was completed to test the differences between EECs of the quadriceps muscle in part I and part II of the study, in the whole group, as well as between LLLT and sham-LLLT interventions. The findings regarding muscle soreness are shown on a T scale. The sham-LLLT was normalised into the actual intervention (LLLT). Biochemical parameters and MVC torques were analysed using two-way ANOVA: 2 (interventions: LLLT and sham-LLLT) multiplied by 6 (time points: baseline, 0, 24, 48, 72, 96 h), with the Bonferroni post-hoc test for multiple comparisons and the values presented as means with standard error (SE). The Statistica v. 12.0 software package was used for calculations.

## Results

### Intensity of NMES and muscle soreness

**Fig. 3:**
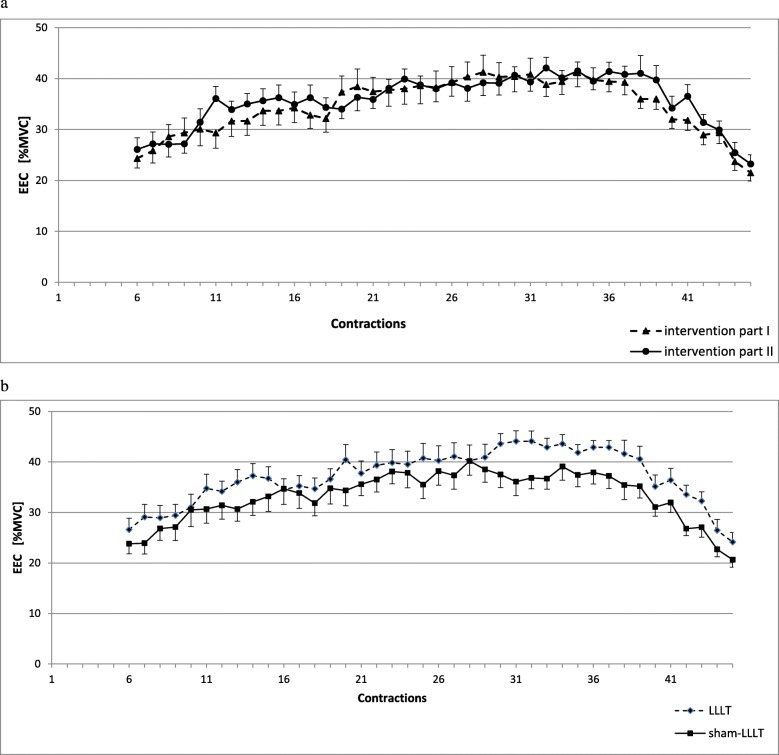
Force tracings of electrically evoked contractions of the quadriceps muscle (from the sixth to the last contraction) (**a**) in the whole group in part I and part II of the study and (B) for electrical stimulation preceded by LLLT irradiations and sham-LLLT interventions. EEC – electrically evoked contractions; MVC – maximal isometric voluntary contraction; LLLT – neuromuscular electrical stimulation preceded by low level laser irradiation; sham-LLLT – neuromuscular electrical stmulation preceded by sham low level laser intervention; values are means ± SE; differences between part I and part II (**a**) as well as between LLLT and sham-LLLT interventions (**b**) were not significant (*p* > 0.05 for group or group and time interaction).

**Fig. 4:**
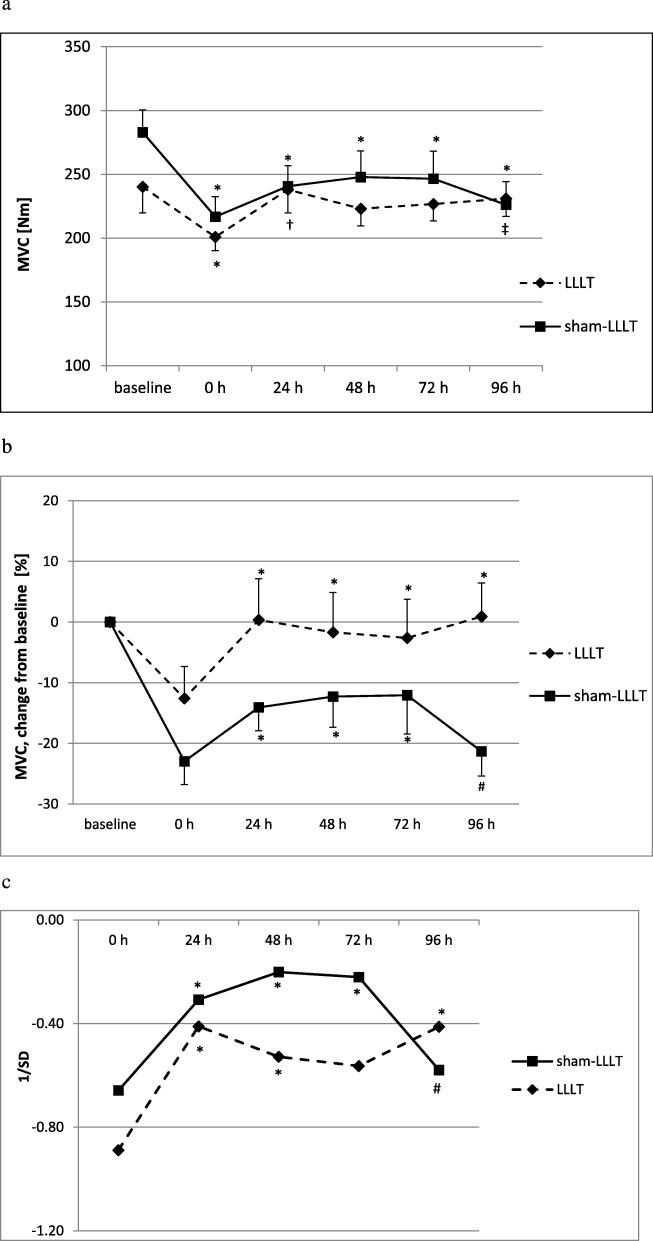
Absolute (**a**) and relative (**b**, **c**) changes in maximal isometric voluntary contractions of the quadriceps femoris muscle following single sessions of neuromuscular electrical stimulation preceded by low-level laser irradiation or sham low-level laser intervention. **b** In percent changes in comparison to baseline. **c** In the values normalized on baseline from two interventions (LLLT and sham-LLLT) combined (Z-score). MVC – maximal isometric voluntary contraction; LLLT – neuromuscular electrical stimulation preceded by low level laser irradiation; sham-LLLT – neuromuscular electrical stmulation preceded by sham low level laser intervention; 0 h – immediately post intervention; 24, 48, 72, 96 h – subsequent hours post intervention; values are means ± SE (**a** and **b**) and means (**c**); **a** main time effect present (*p* < 0.0001); ^*^difference significant (*p* < 0.05), as compared to baseline (within the same intervention); ^†^difference significant (*p* < 0.05) for 24 h, as compared to 0 h (within LLLT intervention); ^‡^difference significant (*p* < 0.05) for 96 h, as compared to 0 h (within LLLT intervention). **b** and **c** main time effect present (*p* < 0.01); ^*^difference significant (*p* < 0.05), as compared 0 h (within the same intervention); ^#^ difference significant (*p* < 0.05) for 96 h, as compared to 48 and 72 h (within sham-LLLT intervention)

The EEC values of the sixth and the last contractions (Fig. 3a) were 24.3 (16.7–36.4) and 21.3 (17.9–33.6) % MVC, and 26.1 (18.6–38.6) and 23.4 (13.4–33.6), in the part I and part II of the study, respectively. The differences between EECs in part I and part II (Fig. 3a) in the whole group, as well as between LLLT and sham-LLLT interventions (Fig. 3b) were not significant (no main effects of group or group and time interaction were observed). The NMES sessions followed with alterations of MVC, with significant time effect (Fig. 4a). In sham-LLLT intervention, a decrease in MVC was seen immediately after NMES (0 h) and it remained decreased, as compared to baseline (*p* < 0.05), throughout recovery period. In turn, LLLT-NMES interventions followed with the decrease in MVC only immediately after NMES (*p* < 0.05 from baseline), then, at 24 and 96 h, MVC increased (*p* < 0.05 from immediately post-NMES). However, no main effects of intervention or time and intervention interaction were observed. Also, only time effect was seen regarding relative changes in MVC, i.e. percentage changes from baseline values of each intervention (LLLT and sham-LLLT) separately (*p* < 0.01; Fig. 4b), or in case of changes in MVC when the values in both LLLT and sham-LLLT interventions were normalised on baseline values from two interventions combined, with standard deviation (Z-score; *p* < 0.01; Fig. 4c).

Muscle soreness following NMES preceded by LLLT and sham-LLLT is presented in Table 3. The differences between the interventions were not statistically significant.

**Table 3:** Muscle soreness following NMES preceded by LLLT and sham LLLT interventions

Intervention	Muscle soreness test	Baseline	post-intervention [hours]				
			0 h	24 h	48 h	72 h	96 h
LLLT	squat test	0	1.1 (0–4.0)	1.4 (0–7.0)	2.0 (0–5.0)	2.5 (0.5–7.0)	1.5 (0–5.8)
	pressure test	0	0.9(0–4.0)	1.4 (0–3.0)	2.2 (0–5.0)	2.4 (0.5–6.0)	1.8 (0–5.5)
Sham-LLLT	squat test	0	1.1 (0–3.5)	1.6 (0–5.0)	2.4 (0–6.0)	2.7 (0–6.8)	1.6 (0–6.0)
	pressure test	0	0.8 (0–4.5)	1.8 (0.5–3.5)	2.7 (1.0–6.0)	2.8 (0–6.0)	1.6 (0–5.0)

Values are means (min value – max value). The differences between the interventions were not statistically significant (*p*

> 0.05)

### Biochemical parameters

The results of redox state parameters are presented in Table 4. No main effects were observed in plasma TAC, and only a tendency in main effects was seen in case of TAC-UA (*p* = 0.08 and *p* = 0.06 for time and time to intervention effects, respectively). Both time and intervention main effects were present in GPx activity in the whole blood (Table 4). No changes in GPx activity were observed after NMES preceded by LLLT. In sham-LLLT intervention, GPx activity decreased at 48 h (*p* < 0.05 from baseline and immediately post-NMES), and increased at 96 h (*p* < 0.05 from baseline, immediately post-NMES and 48 h). At 48 h, the GPx activity was lower in sham-LLLT than in LLLT intervention (*p* < 0.05).

**Table 4:** Changes in redox state and inflammatory markers in response to NMES preceded by LLLT and sham LLLT interventions

variable / biochemical marker	intervention	Parameters at time points of blood sample collections						Main effects		
		baseline	post- intervention [hours]					time [*p* value]	Intervention [p value]	time x intervention [p value]
			0	24	48	72	96			
TAC [mmol/L]	LLLT	1.53 ± 0.04 [0.55]	1.47 ± 0.04 [0.42]	1.42 ± 0.03 [0.35]	1.47 ± 0.04 [0.38]	1.47 ± 0.05 [0.49]	1.50 ± 0.04 [0.48]	0.46	0.50	0.14
	sham-LLLT	1.48 ± 0.05 [0.44]	1.57 ± 0.05 [0.66]	1.49 ± 0.03 [0.26]	1.52 ± 0.04 [0.48]	1.48 ± 0.03 [0.36]	1.45 ± 0.02 [0.29]			
TAC-UA [mmol/L]	LLLT	1.23 ± 0.04 [0.50]	1.16 ± 0.04 [0.45]	1.10 ± 0.03 [0.31]	1.17 ± 0.03 [0.32]	1.14 ± 0.04 [0.32]	1.20 ± 0.03 [0.38]	0.08	0.46	0.06
	sham-LLLT	1.19 ± 0.05 [0.48]	1.26 ± 0.05 [0.61]	1.17 ± 0.03 [0.41]	1.22 ± 0.03 [0.42]	1.15 ± 0.03 [0.29]	1.14 ± 0.01 [0.17]			
GPx [U/g Hb]	LLLT	62.6 ± 2.9^a^ [33.6]	60.8 ± 2.1^a^ [28.1]	61.0 ± 2.8^a^ [38.4]	59.0 ± 3.3^a^ [35.4]	61.5 ± 3.1^a^ [31.6]	63.1 ± 2.1^a^ [26.1]	0.0005	0.044	0.29
	sham-LLLT	56.7 ± 3.8^a^ [42.8]	56.7 ± 2.2^a^ [31.3]	62.7 ± 2.8^ac^ [31.4]	48.3 ± 3.9^b*^ [37.8]	60.4 ± 2.0 ^ac^ [23.5]	65.8 ± 2.9^c^ [29.9]			
SOD U/g Hb]	LLLT	1847.4 ± 160.9^a^ [845.5]	1905.8 ± 157.9^a^ [1322.8]	1985.6 ± 128.9^a^ [1728.0]	1804.2 ± 75.8^a^ [905.8]	1711.3 ± 83.1^a^ [1115.1]	1971.1 ± 137.7^a^ [1552.3]	0.11	0.046	0.87
	sham-LLLT	1821.7 ± 107.8^ab^ [1341.3]	1689.9 ± 152.0^ab^ [1622.7]	1960.5 ± 122.7^a^ [1111.6	1533.8 ± 90.1^b^ [884.1]	1633.3 ± 112.2^ab^ [1369.4]	1774.2 ± 98.1^ab^ [1151.7]			
MDA [μmol/L]	LLLT	1.64 ± 0.52^a^ [5.06]	1.41 ± 0.51^a^ [4.76]	1.16 ± 0.30^a^ [3.45]	2.18 ± 0.72^a^ [7.80]	1.98 ± 0.50^a^ [5.29]	2.04 ± 0.48^a^ [4.91]	0.005	0.72	0.59
	sham-LLLT	1.17 ± 0.17^a^ [1.85]	1.10 ± 0.21^a^ [2.73]	1.49 ± 0.62^ab^ [7.62]	2.40 ± 0.47^b^ [4.47]	2.80 ± 0.68^b^ [6.66]	2.57 ± 0.74^b^ [7.40]			
CRP [mg/L]	LLLT	3.9 ± 0.5^ac^ [5.1]	4.4 ± 0.6^ac^ [6.2]	4.1 ± 0.5^ac^ [5.3]	3.3 ± 0.4^b^ [6.2]	4.9 ± 0.6^a^ [6.6]	3.7 ± 0.4^bc^ [5.4]	0.26	0.33	0.005
	sham-LLLT	3.1 ± 0.5^ab^ [4.6]	3.5 ± 0.6^ab^ [5.0]	3.1 ± 0.4^ab^ [5.8]	3.7 ± 0.5^ab^ [4.9]	3.6 ± 0.5 ^a^ [7.1]	4.7 ± 0.7^b^ [9.5]			

*TAC* Total antioxidant capacity, *TAC-UA* Total antioxidant capacity without uric acid, *GPx* Glutathione peroxidase, *SOD* Superoxide dismutase, *MDA* malondialdehyde, *CRP* C-reactive protein, *TAC* MDA and CRP were measured in plasma; GPx activity were analysed in whole blood; SOD activity was determined in erythrocytes; activities of antioxidant enzymes in erythrocyte and whole blood are expressed in U per gram of haemoglobin, *LLLT* Neuromuscular electrical stimulation (NMES) preceded by LLLT irradiation, *sham-LLLT* Neuromuscular electrical stimulation (NMES) preceded by sham LLLT intervention, *values are means ± SE* With range [max value minus min value from all data within each time point of LLLT or sham-LLLT intervention]

^*^significant difference (*p* < 0.05) between interventions (within the same time point); ^a,b,c^differences between time points (within the same intervention), values that do not have common letters are significantly different (*p* < 0.05)

The activity of SOD in erythrocytes did not change significantly following NMES preceded by LLLT, in contrast to sham-LLLT intervention, where a decrease in SOD activity was present at 48 h (*p* < 0.05 from 24 h; main effect of intervention, *p* < 0.05; Table 4).

Main effect of time occurred for plasma MDA, which increased between at 48 and 96 h after NMES in sham- LLLT intervention (*p* < 0.05 from baseline and immediately post-NMES). However, the LLLT did not affect plasma MDA, because no intervention and time to intervention main effects were observed (Table 4).

Plasma CRP concentration changed differently following LLLT and sham-LLLT interventions, with significant time to intervention effect (Table 4). In LLLT, plasma CRP decreased at 48 h (*p* < 0.05 from immediately post-NMES), then it increased at 72 h (*p* < 0.05 from 48 h), and decreased again at 96 h (*p* < 0.05 from 72 h). In turn, in sham-LLLT intervention, plasma CRP increased significantly at 96 h (*p* < 0.05 from baseline, 0 and 24 h).

**Fig. 5:**
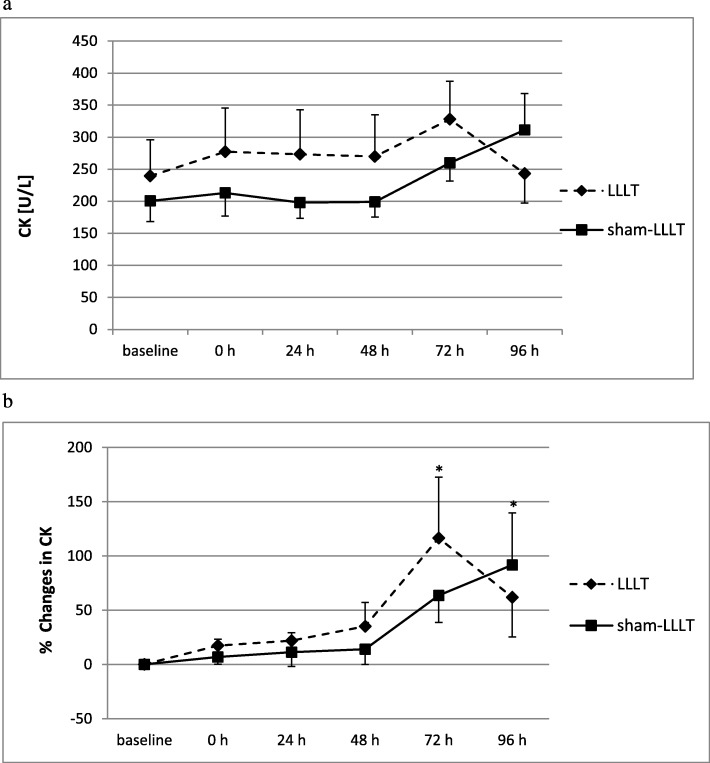
**a** Plasma creatine kinase (CK) activity at baseline and following single sessions of neuromuscular electrical stimulation preceded by single low-level laser irradiation or sham low-level laser intervention. LLLT – neuromuscular electrical stmulation preceded by low level laser irradiation; sham-LLLT – neuromuscular electrical stmulation preceded by sham low level laser intervention; baseline – immediately prior to intervention; 0 h – immediately post intervention; 24, 48, 72, 96 h – subsequent hours post intervention; values are means ± SE; no main effects present (*p* > 0.05); **b** Relative changes in plasma creatine kinase (CK) activity (as compared to baseline) following single sessions of neuromuscular electrical stimulation preceded by single low-level laser irradiation or sham low-level laser intervention. LLLT – neuromuscular electrical stmulation preceded by low level laser irradiation; sham-LLLT – neuromuscular electrical stmulation preceded by sham low level laser intervention; 0 h – immediately post intervention; 24, 48, 72, 96 h – subsequent hours post intervention; values are means ± SE. main time effect present (*p* < 0.05); ^*^difference significant (*p* < 0.05), as compared 0 and 24 h (within the same intervention).

Plasma CK activity did not change significantly in both LLLT and sham-LLLT interventions (no main effects were found for plasma CK activity; Fig. 5a). However, when the relative changes in CK activity were analysed (Fig. 5b), main time effect was observed (*p* < 0.05), with increases in CK activity (as compared to 0 and 24 h) at 72 h after NMES preceded by LLLT and at 96 h after NMES preceded by sham-LLLT.

## Discussion

In the presented study with moderately active, healthy men, a single session of strenuous, isometric NMES of the quadriceps muscle induced oxidative stress, contributing to muscle damage and a decrease in MVC force. The main finding is that LLLT may exert some preventive impact on both antioxidant enzyme system and inflammation responses to a single session of NMES. However, these effects of LLLT on redox state and inflammatory processes do not seem to affect NMES-induced muscle damage, as well as the recovery of muscle function, impaired by NMES.

### Intensity of NMES

We assume that our findings regard a session of strenuous intense NMES. This is confirmed by the range of the measured EEC values in the whole group, typically between 21 and 23% MVC in the last contractions, the subjective perception of the stimulation just below pain threshold in each participant, the observed increase in muscle soreness following the interventions, and the MVC decline in each participant. Electrically evoked contractions of 20–40% MVC are considered as representing intense stimulation [31]. As to the muscle torques after NMES preceded by LLLT, we observed an immediate decrease (from baseline) and subsequent increase (from immediately post-NMES) in MVC until 96 h post intervention. In contrast, NMES preceded by sham- LLLT was followed by significant decreases in MVC (over 20%) immediately post-NMES (as compared to baseline) and 96 h after stimulation (as compared to 48 and 72 h), though with nonsignificant main effect of intervention (*p* > 0.05). The few similar studies correspond, to some extent, with our findings. Jubeau et al. [5] reported a decrease in MVC of up to 25%, lasting several days, while Fouré et al. [10], who used higher frequencies (100 Hz versus 80 Hz in our study), reported a decreased MVC of 29% immediately after NMES.

### Parameters of LLLT

Technical and stimulation parameters of LLLT/ PBMT vary across studies, but our values were within accepted ranges [17, 18, 32]. Also, despite the fact that we designed our study before the publication of proposed recommendations of the parameters of PBMT in exercise performance studies [16], our LLLT parameters are within the clinical recommendations for young adult, healthy participants. Taking into consideration the characteristics and specificity of the applied device, our set of parameters correspond with the recommendations for both technical parameters (light source, power, dose per area, overall dose, wavelength, and mode), irradiation technique (skin contact), as well as for treatment parameters (area and time of irradiations immediately before exercise, as recommended for single events). Therefore, we assume that our findings represent a LLLT/ PBMT technique and dose that allow comparisons with other studies.

### Biochemical response to NMES and LLLT

Immediate decrease in voluntary force production capacities after a single bout of NMES is related to an altered excitation-contraction coupling due to reduced sensitivity of the myofilaments to calcium ions [1, 6]. The increase in circulating CK activity is considered an indirect marker of microdamage within the muscle [33, 34]. We found a significant relative increase in plasma CK activity at 72 h after NMES in LLLT intervention, and at 96 h post-NMES in sham-LLLT condition. This time course observed in our study in sham-LLLT intervention was comparable to previous human studies, applying similar NMES protocols [8–10, 35]. However, in our study, the increase in CK was milder (with average CK activity between 300 and 350 U/L) than in other studies [8–10]. Fouré et al. [10] found a 70 times higher CK level than at baseline at fourth day after NMES. In other studies [8, 9] the increase reached about 1000–1500 U/L at 96 h and was still higher than in our participants. The relatively small increase in CK found in our study might result from higher physical activity of our participants, as supported by our IPAQ assessments, although repeated bout effect cannot be excluded [6, 9]. However, it is worth noting that the studies from Jubeau et al. [5] and Fouré et al. [10] stimulated two legs simultaneously, thereby explaining larger changes in CK activity (30 to 70-fold change), as compared with our study and with Aldayel and colleagues [9]. Also, stimulation parameters, such as higher frequency currents, body position, and muscle length, could all have affected muscle damage profile [1, 6–8, 10]. On the other hand, Zorn et al. [36], similarly to our study, observed only small NMES-induced muscle damage, without muscle soreness, in trained men.

Macrophage infiltration is a histological marker of inflammatory responses following NMES-induced muscle damage [8], with factors such as reactive oxygen and nitrogen species, heat shock proteins and cytokines, involved in muscle degeneration and regeneration [6]. We did not measure cytokines, nonetheless the increase in plasma CK activity was accompanied by the increase in plasma level of CRP at 72 h in LLLT intervention and at 96 h in sham-LLLT intervention. Also MDA concentration increased at 48 h after NMES preceded by sham-LLLT and remained increased until 96 h after NMES. Although we did not measure the levels of reactive oxygen species (ROS) directly, increased plasma MDA as a marker of oxidative damage indicates that a single session of NMES may induce lipid peroxidation, probably as a result of increased production of ROS by inflammatory cells in the process named as the respiratory burst [37].

Inflammatory cells [37, 38] and ROS [39] may contribute to muscle damage but also to its regeneration [6]. However, excessive ROS production may have deleterious effects on structural and functional integrity of cells and tissues. It may deteriorate muscle contractile function and result in muscle fatigue and secondary damage [37]. In our study, the impairment of antioxidant system, observed in sham-LLLT intervention, 48 h after a single session of NMES, might be associated with partial inactivation of enzymatic proteins, resulting from their allosteric or covalent modifications induced by reactive oxygen species. In fact, as suggested by some authors [40, 41], the loss of antioxidant activity after physical exercise may result from exercise-induced oxidative damage to proteins that modify catalytic activity of the enzymes. Therefore, it cannot be excluded that in our study, the increased ROS production following NMES weakened the enzymatic antioxidant system, as indicated by the decrease in SOD and GPX activity at 48 h post sham-LLLT NMES bout. Taken together, the changes in redox state might have affected muscle damage and deterioration in muscle function elicited by a single session of NMES, preceded by sham-LLLT.

Since antioxidant levels are determinants of the regenerative capacity of muscle stem cells, the up-regulation of antioxidant enzymes activity is thought to affect positively muscle repair processes [42]. Based on animal studies, PBMT/ LLLT may stimulate enzymatic antioxidant defence and diminish oxidative damage of lipids after intense exercise [43–45]. On the other hand, increased activity of SOD may reflect enhanced generation of superoxide anions [46]. In accordance with the above, it has even been speculated that PBMT may contribute to a temporary and modest increase in ROS and ATP production and, in turn, the induction of redox sensitive, key regulatory transcription factors, responsible for up-regulation of the activity of antioxidant enzymes [14, 43]. In our study, LLLT preceding a single NMES session also affected the response of enzymatic antioxidant defence (*p* < 0.05 for the effect of intervention).

We observed that both SOD and GPx activities were not altered following LLLT irradiations, in contrast to sham-LLLT condition, where the activities of those enzymes decreased at 48 h post NMES. To the best of our knowledge, this is the first study to report the changes in redox state following an NMES session, as well as the potential of LLLT, to modulate redox state responses to NMES. The apparent protective effect of LLLT on antioxidant enzyme system might result in shortened inflammation period, as indirectly indicated by changes in plasma CRP levels. It decreased at 96 h after NMES preceded by LLLT, in opposite to sham-LLLT condition (*p* < 0.05 for main effect time to intervention). However, measurements of more accurate markers of inflammatory process would test that observation.

Our results also indicate that LLLT does not seem to have protective effect on NMES-induced lipid peroxidation. Plasma MDA was elevated from 48 to 96 h after NMES preceded by sham-LLLT only. However, the differences between the interventions were not statistically significant. Our above mentioned findings extend previous studies, showing an antioxidant effect of LLLT when applied prior to a progressive-intensity running protocol [47], as well as before a futsal match [48]. However, in opposition to those voluntary exercise studies, in our participants the potential preventive effect of LLLT on antioxidant enzymes was not reflected in the time course of muscle recovery, since no significant differences were seen between interventions in CK responses to NMES. Also, our results do not confirm the findings from the only available, pilot NMES clinical study [20], that LLLT irradiations may reduce muscle fatigue and facilitate strength recovery following a fatiguing session of NMES, since no significant main effect of intervention was seen in MVC alterations after NMES. This is perhaps owing to a single NMES session, leading only to a mild increase in plasma CK activity in our physically active, thus adjusted to muscle microdamage, participants. In addition to that, a single LLLT irradiation may not have been sufficient to exert the potential protective mechanisms of LLLT on lipid peroxidation and muscle tissue and, in turn, on muscle function.

### Strengths and limitations of the study

The cross-over design of our study allowed us to gain statistical precision and efficiency that could not be achieved using the parallel design. The intervention effects were tested based on an average of within-individual differences, rather than on a between-group comparisons. These, in turn, reduced the risk of bias in the findings and their interpretation, despite a comparatively small sample size. Nonetheless, the study has limitations. The NMES was strenuous, but we did not receive very similar EECs in each participant, and the subsequent declines in MVC were also varying. This is perhaps due to the nature of NMES, with the contraction force depending on features such as muscle composition, and cross-sectional area, as well as differing distribution of the current. Furthermore, the characteristics of both NMES, and MVC values, dependent on the participants’ perceived pain threshold and their motivation, are therefore prone to some variations, even in the same subjects. The study addressed a short, single intervention in healthy, stable participants. The subjects were already familiar with the NMES intervention and we provided an 8 day the interval between study periods. Nonetheless, we cannot definitely exclude period effects, problems specific in crossover designs, such as the learning effect or the repeated bout effect, as potential confounding factors. As regards external validity, we chose procedures corresponding to other studies [5, 9, 17–20, 32, 35]. Nonetheless, variations across studies in both technical and application parameters of electrical stimulation (e.g. pulse frequency, electrode placement and size), and LLLT (e.g. wavelength, dose and irradiation area), as well as in characteristics of the participants, restrained the generalisability of the findings [15, 17, 18, 32, 43]. Further discrepancies among studies regard differences in NMES procedures of stimulating both legs simultaneously [5, 10] or a single leg [9].

## Conclusion

In conclusion, a single session of strenuous, isometric NMES of the quadriceps muscle in moderately active, healthy men induced oxidative stress, contributing to muscle damage and muscle function impairment. Also, our results suggest the preventive impact of LLLT on antioxidant enzymes as well as its beneficial effects against inflammation, induced by a single session of NMES. On the contrary, the effects of LLLT on redox state and inflammatory processes do not seem to affect muscle damage and recovery of muscle function after NMES. Further studies, especially regarding NMES training programmes with multiple LLLT irradiations, are needed.

## Data Availability

The datasets used and/or analysed during the current study available from the corresponding author on reasonable request.

## Abbreviations

CK: Creatine kinase
CRP: C reactive protein
EEC: Electrically evoked contraction
GPx: Glutathione peroxidase
Hb: Haemoglobin
IPAQ: International Physical Activity Questionnaire
LLLT: Low level laser therapy
MDA: Malondialdehyd
MVC: Maximal voluntary contraction
NMES: Neuromuscular electrical stimulation
PBM: Photobiomodulation
ROS: Reactive oxygen species
SOD: Superoxide dismutase
TAC: Total antioxidant capacity
TAC-UA: total antioxidant capacity without uric acid
UA: uric acid
